# Which is most important for mental health: Money, poverty, or paid work? A fixed-effects analysis of the UK Household Longitudinal Study

**DOI:** 10.1016/j.ssmph.2021.100909

**Published:** 2021-09-04

**Authors:** Theocharis Kromydas, Rachel M. Thomson, Andrew Pulford, Michael J. Green, S. Vittal Katikireddi

**Affiliations:** a MRC/CSO Social & Public Health Sciences Unit, University of Glasgow, United Kingdom; b Public Health Scotland, United Kingdom

**Keywords:** Mental health, Income, Poverty, Employment, Welfare, Health inequalities

## Abstract

**Background:**

The relative importance of income, poverty and unemployment status for mental health is unclear, and understanding this has implications for income and welfare policy design. We aimed to assess the association between changes in these exposures and mental health.

**Methods:**

We measured effects of three transition exposures between waves of the UK Household Longitudinal Study from 2010/11–2019/20 (n=38,697, obs=173,859): income decreases/increases, moving in/out of poverty, and job losses/gains. The outcome was General Health Questionnaire (GHQ), which measures likelihood of common mental disorder (CMD) as a continuous (GHQ-36) and binary measure (score ≥4 = case). We used fixed-effects linear and linear probability models to adjust for time invariant and time-varying confounders. To investigate effect modification, we stratified analyses by age, sex and highest education.

**Results:**

A 10% income decrease/increase was associated with a 0.02% increase (95% CI 0.00, 0.04) and 0.01% reduction (95% CI -0.03, 0.02) in likelihood of CMD respectively. Effect sizes were larger for moving into poverty (+1.8% [0.2, 3.5]), out of poverty (−1.8%, [-3.2, −0.3]), job loss (+15.8%, [13.6, 18.0]) and job gain (−11.4%, [-14.4, −8.4]). The effect of new poverty was greater for women (+2.3% [0.8, 3.9] versus +1.2% [-1.1, 3.5] for men) but the opposite was true for job loss (+17.8% [14.4, 21.2] for men versus +13.5% [9.8, 17.2] for women). There were no clear differences by age, but those with least education experienced the largest effects from poverty transitions, especially moving out of poverty (−2.9%, [-5.7, −0.0]).

**Conclusions:**

Moving into unemployment was most strongly associated with CMD, with poverty also important but income effects generally much smaller. Men appear most sensitive to employment transitions, but poverty may have larger impacts on women and those with least education. As the COVID-19 pandemic recedes, minimising unemployment as well as poverty is crucial for population mental health.

## Highlights

• Mental health impacts of income change are largely explained by poverty transitions.
• Job losses or gains are more important for mental health than income or poverty.
• Women's mental health may be more sensitive to poverty.
• Contrastingly, men's mental health appears more sensitive to employment.
• The least educated appear most sensitive to poverty changes.

## 1 Introduction

People living on lower incomes, particularly those living below the poverty line, are more likely to have poor mental health and wellbeing (Kessler et al., 1994; Marmot, 2005; Ngui et al., 2010; Subramanian & Kawachi, 2006). A similar cross-sectional association exists between unemployment and poor mental health (Ohayon et al., 1999). However, while longitudinal and natural experiment studies frequently find job loss is followed by a worsening in mental health (Milner et al., 2014; Paul & Moser, 2009), adding weight to this potentially being a causal phenomenon, there is less consensus on whether the same is true for income losses or gains, especially in high-income countries (Chandra & Vogl, 2010). In fact, the impression from longitudinal income change studies is of a surprisingly small effect size on health after controlling for key confounders, if one exists at all (Mackenbach, 2020). However, there are some important additional factors to consider before drawing the conclusion that money itself is not important for mental health.

Firstly, changes in employment status are unlikely to occur without concurrent changes in income, and the relative contribution of each of these factors is often not actively considered when measuring the impact of job loss on mental health (Milner et al., 2014; Paul & Moser, 2009). Secondly, the effects of income and employment changes on mental health may be non-reciprocal: income losses may have a greater magnitude of effect than income gains (Boyce et al., 2013) and the positive effects of re-employment may be larger than the negative effects of job losses (McKee-Ryan et al., 2005). Thirdly, there is evidence that there may be a specific threshold effect on mental health and wellbeing of moving above or below a key level of absolute income such as the poverty line (Dang et al., 2019; McCarthy et al., 2018; Wickham et al., 2017). However, this has typically been studied as a separate binary exposure, rather than alongside consideration of income changes on a continuous scale.

Finally, a seemingly unconvincing relationship between income changes and mental health at a population level may mask differential impacts in population subgroups, particularly for those of lower socioeconomic position (SEP) (Owusu-Addo et al., 2018; Simpson et al., 2021). It is plausible that there is both a threshold effect *and* effect modification by SEP, i.e. that there is a level of income which offers sufficient protection against material deprivation and the negative wellbeing consequences of this (the poverty line), but also a differential effect of income according to SEP over and above this (Cooper & Stewart, 2015). Considering other potential effect modifiers such as sex, Barbaglia et al. find that while job losses impact on three-year incidence of mental disorders in men but not women, the opposite is true for reductions in household income (Barbaglia et al., 2015). Few studies in this field stratify by age as opposed to simply adjusting for this as a potential confounder, but it is known from literature on the introduction of austerity policies that those in younger working-age groups potentially experienced greater mental health impacts (Thomson & Katikireddi, 2018).

Drawing from this evidence base, we therefore hypothesise that:

A. The impact of changes in employment status on mental health may be partially explained by concurrent changes in income;
B. The direction of income and employment changes may impact on the magnitude of the effect on mental health;
C. The average effect of income change on mental health may be less than the effect of transitions into or out of poverty;
D. Income changes may affect mental health more for those of lower SEP (D1), for women (D2), and for those in younger age groups (D3).

While several studies have explored the relationship between absolute income change and mental health using UK panel data (e.g. (Boyce et al., 2013; Mendolia, 2014)), as far as we are aware none have simultaneously considered the specific contribution of moving above or below the poverty line and concurrent employment transitions, or the role of the potential effect modifiers described above. Jones and Wildman come closest to this by including a broad measure of relative deprivation in their analyses (Jones & Wildman, 2008), but do not consider income increases and decreases separately and do not investigate differing effects by subgroup. Therefore, using fixed-effects analysis of a representative UK panel, we investigated the separate and combined effects of income, poverty, and employment transitions on mental health in the working-age population, and explored potential effect modification by age, sex, and SEP.

## 2 Material and methods

### 2.1 Data

We used data from the representative UK Household Longitudinal Study (UKHLS), also referred to as Understanding Society (hereafter abbreviated as USoc), which includes around 40,000 households from 2009 onwards (University of Essex and Inst, 2020). Data were collected annually on all adults aged 16 years or over within included households, either by in-person interview or via an online self-completion questionnaire. Data were available from ten waves of USoc, the last of which was completed in 2019 with a small number of observations from early 2020.

### 2.2 Population

We restricted analyses to waves 2 to 10 (2009/10 to 2019/20) because of our focus on income and employment transitions between two consecutive waves. We included all individuals who participated in two consecutive waves at least once.

### 2.3 Exposure measurement and covariates

Drawing on the concepts and context outlined in the introduction, we estimated five effects of income, and three of employment status: first, the effects of household income change (distinguishing increases from decreases); second, separate effects for transitioning into, out of, or remaining in poverty; and third, separate effects for transitioning into, out of, or remaining in unemployment on mental health. We used OECD equivalised household income after deducting housing costs, to ensure comparisons were valid between different geographies and to account for changes in the housing market or mortgage interest rates during the study period. Income was adjusted for inflation using the average inflation figure for the relevant years in each wave. Non-missing extreme values (p > 0.001) were replaced in both tails of the income distribution with the next values counting inwards from the extremes (Winsorisation).

Estimates for **income increases** and **income decreases** were derived by a two-way interaction between a binary variable that showed whether income had increased or decreased between two consecutive waves, and a continuous variable that showed the absolute value of the amount of this change. Finally, these variables were log-transformed using the natural logarithm to account for data skewness and linearise the relationship with the outcome variable.

Poverty was represented by a binary variable based on the poverty threshold, which was defined as 60% of the median household equivalised income after housing costs to reflect the Households Below Average Income (HBAI) measures for relative poverty (Department for Work &, 2020). In total three poverty effects were calculated as a result of income change between two consecutive waves: one for moving below the poverty line (**into poverty**), another one for moving above the poverty line (**out of poverty**), and a third for remaining below the poverty line (**persisting poverty**). Throughout all analyses, the reference category for the ‘into poverty’ and ‘persisting poverty’ groups was those who remain out of poverty in both waves, whereas the ‘out of poverty’ group is compared with those who remain in poverty in both waves.

To calculate employment transitions, we first recoded the original USoc employment status variable to reflect four broader employment status categories: Employed (including employees, the self-employed, those on maternity leave and those working for unpaid family businesses), Unemployed, Inactive (including full-time students, those who are retired, those providing family care at home, those in governmental training schemes, and those on apprenticeships or similar) and Long-term sick and disabled. Although all transitions among these categories were calculated, our primary focus was on the effect of transition from being employed to being unemployed (**into unemployment**), from being unemployed to being employed (**out of unemployment**), and of remaining unemployed in both waves (**persisting unemployment**). As with the poverty transitions, the first and third effects were analysed relative to remaining in employment, while for the second effect the reference category was persisting unemployment.

For better model identification purposes, we used one-wave lagged terms for time-varying control variables to minimise the potential for reverse causality to influence results. Confounders from the current wave included age as a continuous variable along with its squared term, educational level in four broad categories (Degree/Other, A-Level, GCSE, None/Other) and government office region, with one-wave lagged terms used for mental health, physical health, household structure, household income (log-transformed to match our income change exposure variable), whether any member in the household received any benefits (yes/no) and whether household income was increased by any gains from savings/investments (yes/no). Education was selected as this can be considered a measure of early adulthood SEP which remains largely static after that point in the life course, in contrast with our exposure variables of income and poverty status which are more fluctuant, thus capturing a different dimension of SEP (Galobardes et al., 2006; Geyer et al., 2006).

### 2.4 Outcome measurement

We measured mental health using the General Household Questionnaire (GHQ), a commonly used screening tool in epidemiological literature for symptoms of probable common mental disorder (CMD) e.g. anxiety/depression (Goldberg & Williams, 1988). We included a continuous outcome measure, derived from the GHQ-36 item Likert scale (with higher scores indicating poorer mental health), and binary indicators derived from the GHQ-12 item scale dichotomised in two ways. In our main analysis (Specification 1), individuals with a GHQ-12 score ≥4 were identified as having CMD. For sensitivity purposes, we included an additional analysis (Specification 2) which lowered the cut-off for CMD to GHQ-12 score ≥3, as this threshold is also commonly used in existing literature.

### 2.5 Statistical analysis

We first conducted descriptive analyses to describe prevalence of CMD for each wave stratified by our exposures of interest. To estimate the effect of our three transition types of interest on mental health, we then conducted a weighted two-level (individual and waves) fixed-effects model for panel data. We calculated a non-response non-monotone weight to account for attrition and the fact that our main exposures refer to transitions. The weights were modified to account for missing data in our outcome variable, with a sensitivity analysis run without this adjustment to investigate the impact of item missingness on results. We used fixed-effects linear regression for our continuous GHQ-36 variable and a linear probability model (LPM) for our binary outcome variables representing likely CMD. We selected an LPM over fixed-effects logistic regression to ensure we retained sufficient sample sizes for our stratified analyses, but performed sensitivity analysis comparing the LPM with a conditional logit model to ensure this choice did not affect results. Further details about our modelling specification and weighting strategy are included in Appendix A.

For our unstratified analysis we estimated a series of two-way fixed-effect models for: (a) income change variables, (b) poverty transition variables, and (c) employment transition variables. Firstly, “Model 1” consisted of a group of three unadjusted linear models with GHQ-36 as the outcome variable. Model 1a included the interaction described in section 2.3 between the binary variable that showed whether log income had increased or decreased with the amount of that change. Models 1b and 1c performed the same function for the poverty transition and employment transition variables respectively. Secondly, Models 2a, 2b and 2c added all control variables outlined in section 2.3 to these models, except from the other two transition exposures of interest in our study. Finally, Model 3 included all three sets of transition exposures in a single model to identify how the effects changed when our variables of interest were mutually adjusted. Models 3* and 3** were LPMs with presence/absence of likely CMD as their outcome variable (with 3* using cut-off of GHQ ≥4 and 3** GHQ ≥3 as discussed above). Models 1 and 2 used our tailored non-response weight which took into account the likelihood of attrition between two consecutive waves, and therefore our sample is restricted to individuals with no missing data for all our key variables to allow comparison across nested models.

In addition to our main unstratified analysis, we also estimated stratified models to investigate whether the effect of income change or poverty/employment transitions differed by sex, age group (18-29y, 30-44y and 45-65y) or, as a proxy for SEP, highest educational attainment (Degree/Other Higher, A-Level. GCSE, None/Other). For ease of interpretation, in all tables we report the effects of income changes as 10% increases or decreases, rather than as the beta coefficient for the logged variable.

## 3 Results

### 3.1 Descriptives

From the initial USoc sample of 87,045 people (444,181 observations) we excluded those who did not complete a full interview (N = 5170), those who were not aged 18–65 years (N = 14,381), those with no valid weights (N = 28,350) and those with missing values for any variable of interest (n = 4778). Our final analytical sample therefore included 38,697 people across 173,859 observations, 49.9% of whom participated in at least seven of the included waves (see Appendix B for detailed flowchart and Table C1 in Appendix C for full details on participation).

Table 1 presents descriptive statistics for the exposure and outcome variables as well as all covariates used in our Models (additional detail on continuous variables is included in Appendix Table C2). For the income change exposure, 43.6% of our observations captured an income increase compared with the previous year, with 56.4% capturing an income decrease; the mean value of income decreases was higher than increases (£6999.86 versus £5906.56). Transitions into poverty occurred in 8.3% of our observations, with 8.7% recording a transition out of poverty. For unemployment, 1.3% of our observations captured a move into unemployment, and 1.7% a move back into employment. The mean value of our continuous GHQ-36 measure across the sample was 11.3 (SD 5.6), and the prevalence of likely common mental disorder across all observations was 19.5% using a cut-off of GHQ ≥4 and 23.9% using a cut-off of GHQ ≥3.

**Table 1:** Number of observations and individuals in all categorical variables used in unstratified regression models.

Outcome	Variable	Value	Observations	Mean	Individuals	Std.Dev
	**Outcome as Continuous**	GHQ-36 (Min = 0, Max = 36)	173,859	11.29	38,697	5.63
	**Variable**	**Value**	**Observations**	**%**	**Individuals**^a^	**%**
	**Specification 1 (GHQ ≥ 4)**	No common mental disorder	140,014	80.5	35,718	92.3
		Common mental disorder	33,845	19.5	15,772	40.8
	**Specification 2 (GHQ ≥ 3)**	No common mental disorder	132,299	76.1	34,851	90.6
		Common mental disorder	41,560	23.9	18,317	47.3
**Exposures**	**Income change**	Income decrease	75,883	43.6	31,652	81.8
		Income increase	97,976	56.4	34,400	88.9
	**Poverty**	No poverty both waves	118,032	67.9	30,227	78.1
		Into poverty	14,443	8.3	11,484	29.7
		Out of poverty	15,045	8.7	11,976	31.0
		Persisting poverty	26,339	15.2	10,868	28.1
	**Economic Activity**^b^	**Employment both waves**	**120,235**	**69.16**	**27,842**	**71.95**
		**Employment to Unemployment**	**2274**	**1.31**	**2154**	**5.57**
		Employment to inactivity	4518	2.6	4236	10.95
		Employment to Long-Term disability	479	0.28	466	1.2
		**Unemployment to Employment**	**2882**	**1.66**	**2693**	**6.96**
		**Unemployment in both waves**	**3826**	**2.2**	**2104**	**5.44**
		Unemployment to Inactivity	1595	0.92	1473	3.81
		Unemployment to Long-term disability	662	0.38	592	1.53
		Inactivity to Employment	4238	2.44	3891	10.06
		Inactivity to Unemployment	1618	0.93	1496	3.87
		Inactivity in both waves	23,961	13.78	9548	24.67
		Inactivity to Long-Term disability	638	0.37	568	1.47
		Long-term disability to Employment	286	0.16	281	0.73
		Long-term disability to Unemployment	565	0.32	503	1.3
		Long-term disability to Inactivity	879	0.51	778	2.01
		Long-term disability in both waves	5203	2.99	1796	4.64
**Confounders**	**No of children< 16y (t-1)**	No children	134,427	77.3	32,333	83.6
		One child	17,868	10.3	6341	16.4
		Two children	15,643	9.0	4705	12.2
		Three or more	5921	3.4	1754	4.5
	**Educational level**	Degree/Other Higher	75,472	43.4	15,734	40.7
		A-Level	38,522	22.2	9887	25.6
		GCSE	35,933	20.7	8457	21.9
		None/Other	23,932	13.8	6369	16.5
	**Household structure (t-1)**	Coupled household with children	52,367	30.1	13,977	36.1
		Coupled household, no children	41,905	24.1	11,907	30.8
		Multiple adults ± children	43,324	24.9	15,091	39.0
		Single female	13,714	7.9	3877	10.0
		Single male	12,493	7.2	3485	9.0
		Single parent	10,056	5.8	3488	9.0
	**Benefit status (t-1)**	No	64,200	36.9	18,565	48.0
		Yes	109,659	63.1	30,403	78.6
	**Savings status (t-1)**	No	117,271	67.5	33,636	86.9
		Yes	56,588	32.5	18,669	48.2
	**Region**	North East	6902	4.0	1492	3.9
		North West	18,280	10.5	4139	10.7
		Yorkshire and the Humber	13,790	7.9	3165	8.2
		East Midlands	13,728	7.9	3090	8.0
		West Midlands	13,812	7.9	3176	8.2
		East of England	15,408	8.9	3452	8.9
		London	17,273	9.9	4550	11.8
		South East	21,772	12.5	4889	12.6
		South West	14,985	8.6	3184	8.2
		Wales	11,692	6.7	2757	7.1
		Scotland	15,557	8.9	3485	9.0
		Northern Ireland	10,660	6.1	2579	6.7
	**Full sample**		**173,859**		**38,697**	

a This shows the number of individuals who have been in each category at least once, so percentages in the next column do not sum to 100.

b The employment transitions of interest are in bold.

**Fig. 1:**
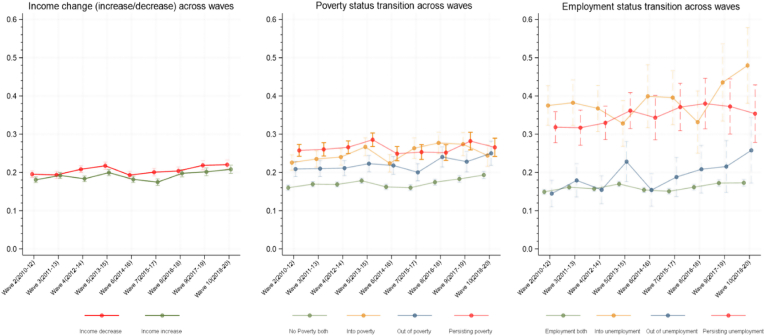
Prevalence of likely common mental disorder (CMD) stratified by income change, poverty, and employment status.

Fig. 1 shows the prevalence of CMD for each of our exposure groups across all included waves of USoc. From the graph it appears that income changes on average were not strongly correlated with CMD, and there was no clear difference between income increases and decreases. Regarding poverty status transitions, all three groups who experienced either recent or current poverty appear more likely to have experienced CMD than those who remained out of poverty, with those in persisting poverty having the highest prevalence. For employment status transitions there is an even clearer trend, where those who experienced unemployment (either new or persisting) reported higher prevalence of CMD compared to those who did not.

### 3.2 Main analyses: the effect of employment status, poverty and income changes

Table 2 shows the effect of all our exposures on GHQ-36 score and likelihood of CMD. For better illustration purposes, this effect is also illustrated visually in Figures D1 and D2 in Appendix D.

**Table 2:**
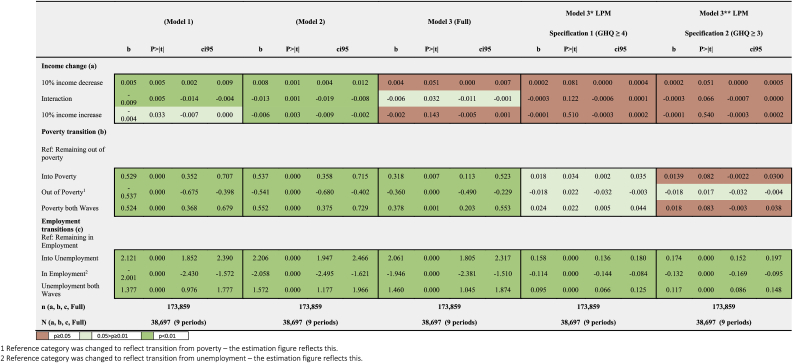
Effects of income, poverty, and employment transitions on GHQ-36 score (Models 1,2,3) and likelihood of Common Mental Disorder (Models 3* and 3**)..

#### 3.2.1 Job losses and gains

The effect of employment status transitions and persisting unemployment were the largest among all estimated exposures’ effects. Looking at Model 3 (which is mutually adjusted for the other two exposure variables), moving into unemployment seemed to have a large and negative effect on mental health (worsening of 2.061 GHQ points [95% CI: 1.805, 2.317]). Comparing across the three models, the effect sizes for employment transitions were slightly larger in Models 1 and 2 compared to Model 3, in support of Hypothesis A (that income changes likely explained some of the effect of employment transitions on mental health). However, the reduction in effect magnitude in the adjusted models was less notable than with the other two exposures, which may indicate that unemployment is a stronger independent determinant of mental health compared to poverty or income.

Moving into unemployment had the largest effect among all employment status transitions for our continuous outcome measure, supporting Hypothesis B (that the direction of the transition was likely to be important for effect magnitude). The probability of having a CMD for someone who moved into unemployment increased by 15.8% [95% CI: 13.6%, 18.0%], which was larger than the 11.4% decrease [95% CI: −14.4%, −8.4%] in the same probability for someone who moved out of unemployment. Persisting unemployment had a large effect, but it was smaller than the other two transitions: this was the case for both the linear model (worsening of 1.460 GHQ points [95% CI: 1.045, 1.874]) and the LPM [9.5% increase in likelihood of CMD [95% CI: 6.6%, 12.5%]]. The effect of the other economic activity transitions calculated can be found in the Appendix (Table C3).

#### 3.2.2 Moving in and out of poverty

In support of Hypothesis C, the effects for poverty status transitions and persisting poverty were considerably larger than those for income change, but similarly to the findings for employment transitions the magnitude of effect for each was larger in Models 1 and 2 compared to mutually adjusted Model 3. All three poverty status effects are of a similar magnitude, indicating Hypothesis B did not hold for poverty transitions. This implies that, on average, the short-term effect of moving into poverty on mental health (worsening in mental health of 0.318 GHQ points [95% CI: 0.113, 0.523]) could be reversed by moving out of poverty (improvement of 0.360 GHQ points [95% CI: −0.490, −0.229]). Moreover, persisting poverty did not seem to have a greater effect than moving into poverty, with its effect slightly smaller (worsening of 0.378 points [95% CI: 0.203, 0.553]). Estimates from the LPM indicated that moving out of poverty was related to a decrease in the probability of having CMD of 1.8% [95% CI: −3.2%, −0.3%] using Specification 1 (GHQ ≥4), with Specification 2 (GHQ ≥3) finding very similar results. The magnitude of effect was relatively similar for both moving into poverty (1.8% increase in likelihood of CMD [95% CI: 0.02%, 3.5%]) and persisting poverty (2.4% increase in likelihood [95% CI: 0.5%, 4.4%]).

#### 3.2.3 Income losses and gains

The effect sizes for log income increases and decreases were very small, and became increasingly weaker after adjustment for confounding variables. As with poverty Hypothesis B was not supported, as there was no evidence of a marked difference in the effect between income increases and decreases. In the mutually adjusted Model 3, a 10% income decrease was associated with a decline in mental health of 0.004 [95% CI: 0.000, 0.007] points on the GHQ-36-point scale, while a 10% income increase improved mental health by 0.002 [95% CI: −0.005, 0.001] points. Estimations from the LPM model provided similar results for both specifications, with very weak effects.

### 3.3 Stratified analyses: differential effects by sex, age and socioeconomic position

#### 3.3.1 Differences by highest educational attainment

In support of hypothesis D1, the mental health of the least educated individuals was more likely to be affected by poverty transitions and persisting poverty compared to all other educational groups (Table 3) e.g., for moving out of poverty there was a 0.689 point improvement in GHQ score for the lowest educated [95% CI: −1.045, −0.332] versus 0.264 GHQ points for A-level educated [95% CI: −0.675, 0.148]. Similarly, looking at the employment transitions the effect of job loss was the highest for the least educated group (worsening of 2.390 GHQ points [95% CI: 1.413, 3.366]). However, the A-level educated group appeared particularly sensitive to job gains when considering likelihood of CMD, with this improving by 17.7% [95% CI: −27.0%, −8.5%]. Again, job loss seemed to have the biggest impact of the employment status transitions for all groups except those with A-level education.

**Table 3:**
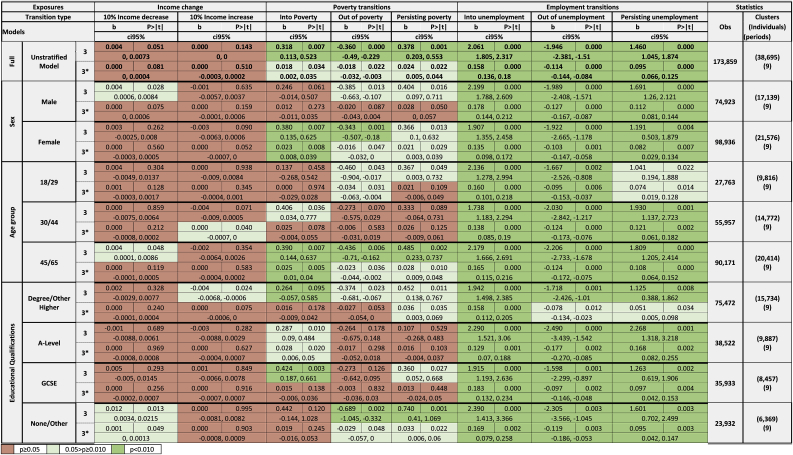
Effects of income, poverty and employment transitions on GHQ-36 score (Model 3) and likelihood of Common Mental Disorder (Model 3*) stratified by sex, age and education..

#### 3.3.2 Differences by sex

Stratifying our income change analyses by sex did not result in any notable difference to our estimations, and these remained very small. In support of Hypothesis D2, the effect of moving into poverty was stronger for women (worsening of 0.380 GHQ points [95% CI: 0.135, 0.625] and 2.3% increase in likelihood of CMD [95% CI: 0.8%, 3.9%]) compared to men (worsening of 0.246 GHQ points [95% CI: −0.014, 0.507] and 1.2% increase in likelihood of CMD [95% CI: −1.1%, 3.5%]), albeit both were imprecisely estimated. The differences between the two sexes for the other two poverty status transitions did not substantially differ. For employment effects there were some differences by sex, with men seeming to be more sensitive to all employment status transitions e.g. job loss was associated with a 17.8% increase in likelihood of CMD for men [95% CI: 14.4%, 21.2%] versus 13.5% for women [95% CI: 9.8%, 17.2%] – see Table C4 and Figure D2 for Specification 2 using GHQ cut-off 3+. All sex-stratified coefficient estimates are illustrated in Figures D3 and D4 in Appendix C (as are those stratified by age group/highest educational attainment).

#### 3.3.3 Differences by age

Once more, the effect size for income change remained very small across all subgroups, and age-stratified findings for the other exposures were fairly inconsistent. Hypothesis D3 (that younger groups would experience larger effects of income changes) was not clearly supported. Moving out of poverty seemed to affect the mental health of the youngest group more than the middle group (improvement of 0.460 GHQ points [95% CI: −0.904, −0.017] for 18–29 year olds versus 0.273 GHQ points [95% CI: −0.575, 0.029] for 30–44 year olds], though the opposite was true for moving into poverty. The oldest group (aged 45–64 years) had large effect sizes for all poverty transitions. The effect of moving into unemployment seemed strong for all age groups but was slightly larger for the oldest group [2.125 for 45–65 year olds; 95% CI: 1.710, 2.539 versus 2.062; 95% CI: 1.368, 2.738 for 18–29 year olds]. Those aged 30 to 44 seemed most sensitive to persisting unemployment (worsening of 1.930 GHQ point [95% CI: 1.127, 2.723]), whereas the oldest group seemed most sensitive to job gains (improvement of 2.206 GHQ points [95% CI: −2.733, −1.678]) and job losses (worsening of 2.179 GHQ points [95% CI: 1.666, 2.691]). As with sex, results from LPMs indicate that for all age groups new unemployment increased the probability of CMD more than persisting unemployment, and also more than a job gain decreased it.

### 3.4 Summary of effects

**Fig. 2:**
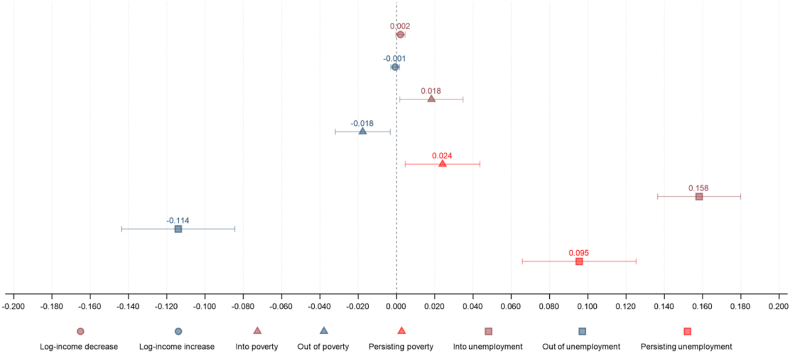
Summary of the impact of each exposure of interest on likelihood of common mental disorder, after adjustment for all confounders (Model 3*).

Finally, to illustrate the relative magnitude of each effect of interest and to highlight the potential impact on burden of disease, Fig. 2 shows the effect of each exposure grouping on the likelihood of CMD after adjustment for all confounders (using specification 1, GHQ ≥4).

## 4 Discussion

### 4.1 Key findings

In this representative cohort of the UK working-age population, after accounting for individual and wave fixed-effects and important confounding variables we find very small effect sizes for the relationship between income changes and mental health. However, we find larger effects on mental health where individuals cross the poverty line or move into and out of employment, with these transitions more likely to have important impacts on prevalence of common mental disorder (CMD) at a population level (Fig. 2). The impact of poverty transitions on GHQ-36 score is larger than the effect of income change, but still only appears to result in at most a 1–2% change in the likelihood of experiencing a CMD. In contrast, the effect of job loss or gain is considerably larger and is associated with a 15.8% increased or 11.4% decreased likelihood in having a CMD respectively even after taking into account concurrent income changes. In contrast, the relationship between income change and mental health is attenuated when poverty and employment transitions are accounted for within the analysis.

We find no notable difference in the effect of income changes across subgroups by age, sex or socioeconomic position (as measured by educational attainment), though this is not unexpected given the weakness of this association in the whole population: with only a few exceptions, it remains consistently very weak with relatively large confidence intervals (Table 3). The effect of moving across the poverty line on mental health appears to be strongest for those with the least education, with a move out of poverty associated with a 2.9% [95% CI: 0.0%–5.7%] reduction in likelihood of CMD for those with no formal qualifications. Women seemed more sensitive to moving into poverty than men, with likelihood of CMD increasing by 2.3% [95% CI: 0.8%–3.9%) versus 1.2% for men [95% CI: −1.1%–3.5%], though men were affected more than women for all employment status transitions. Job losses had consistently larger effects than job gains across almost all subgroups.

### 4.2 Our findings in context

Our lack of evidence for a strong causal effect of income change on mental health is not out of keeping with existing literature in this area (Chandra & Vogl, 2010; Mackenbach, 2020), which is itself similar to the literature on income changes and general health [e.g. Frijters et al., 2005]. It has been known for some time that as levels of income within a country rise over time, the health and wellbeing gains that might be expected from the strong cross-sectional income/health gradient do not appear (Easterlin, 1995). This phenomenon, known as the Easterlin Paradox, has led economists to debate whether a causal relationship between absolute income and wellbeing exists at all after confounding is accounted for (Frey & Stutzer, 2002).

Studies which consider within-person changes in household income using similar methods to ours have typically found no or very small effects on mental health or wellbeing outcomes after adjustment for important confounders such as employment status, marital status and past mental health (Binder & Ward, 2013; Jones & Wildman, 2008). Similar small effect sizes are seen when considering the impact of more plausibly exogenous income shocks such as lottery wins or unexpected windfalls (Apouey & Clark, 2015; Cesarini et al., 2016). It has been argued that, at least in the context of subjective measures such as happiness and life satisfaction, changes in relative income in comparison with others or a person's past may in fact be more important than absolute income alone (Clark et al., 2008). Our replication of the findings of others (Dang et al., 2019; McCarthy et al., 2018; Wickham et al., 2017) in relation to the importance of the poverty line in this context – which is by definition a marker of relative position in society as well as a measure of material deprivation – potentially adds weight to this theory, particularly given that we take care to simultaneously adjust for absolute income changes.

In a 2015 review by Cooper and Stewart for the charitable organisation the Joseph Rowntree Foundation, the authors bring together data solely from experimental or longitudinal studies which could plausibly be interpreted in a causal way and argue that there is sufficient evidence for a causal effect of income change on mental health outcomes (Cooper & Stewart, 2015), despite effect sizes being relatively small. The studies which they report having the largest effects are among those targeted at specific vulnerable populations such as parents on low incomes, [e.g. Gennetian and Miller, 2002] one of which reports a similar finding to ours of an enhanced effect when an income change moved a household above the poverty line (Dearing et al., 2004). This is in keeping with our Hypothesis C of there being both a potential threshold effect as well as differing effects of income changes at different levels of SEP, which was borne out in our analysis of poverty transitions if not income changes alone. Interestingly, when the authors recently published a similar systematic review focusing on childhood outcomes (Cooper & Stewart, 2020), they specifically reported that studies using experimental and quasi-experimental methods tended to find larger effect sizes than fixed-effects studies.

While income change studies do not routinely differentiate between increases and decreases, there was some pre-existing evidence that income losses may have slightly larger effects on mental health than income gains at a population level (Boyce et al., 2013). In our study we found that the effect of moving above the poverty line does not seem to have a considerably larger positive impact on mental health than the corresponding negative effect resulting from moving below it for our continuous outcome measure. Regarding unemployment and mental health, existing literature indicates that re-employment may have a larger magnitude of effect than job loss (McKee-Ryan et al., 2005), though Paul and Moser attribute this apparently paradoxical finding to issues with repeated testing (Paul & Moser, 2009). In contrast, we find in our sample that the effect of job loss appears to be consistently slightly larger than that of job gain.

Our stratified findings are largely in keeping with what was expected from the existing literature – for example, the greater negative effect of crossing the poverty line or remaining in poverty for those with least education (Cooper & Stewart, 2015) and the impact of job loss and gain being greater for men (Paul & Moser, 2009). In particular the finding that job loss appears to be more harmful for male than female mental health is well established, and has been attributed both to increased stigma associated with male unemployment (McFadyen, 1995) and work being particularly central to masculine identity (Kulik, 2000). Our finding of a sex-related difference between the impact of moving into poverty on the likelihood of CMD partially confirms that of Barbaglia et al. who found that household income losses appeared to be a more important contributor to development of mental disorders for women than men in a Dutch cohort (Barbaglia et al., 2015). With the exception of Dang et al. who report a similar relationship to Barbaglia (Dang et al., 2019), the other studies we are aware of which specifically consider a binary poverty threshold either restrict their sample to only women (Wickham et al., 2017) or adjust for sex rather than stratifying their results (McCarthy et al., 2018). Given the lack of evidence, better understanding the gender differences in response to poverty transitions may be a useful area for future research.

Lastly, it is important to acknowledge the exact nature of the exposure and outcome we are studying in our analyses. By focusing only on transitions between survey waves we are considering a fairly narrow concept of the effect of income, poverty or unemployment on mental health, occurring only in the short to medium-term. We cannot be sure how poverty and employment status change within the interim period between two consecutive waves, particularly for those with income levels marginally below or above the poverty threshold and for those in precarious employment. While this exposure does have important policy relevance, it means by definition we are not taking into account any effect of wealth or day-to-day financial insecurity (Niedzwiedz et al., 2017), or any pervasive or cumulative effects of living in poverty or on a low income (Lynch et al., 1997).

### 4.3 Strengths and limitations

A key strength of our study is the use of a representative sample of the UK population followed over a long time period, allowing us to observe a considerable number of the transitions in which we are interested. We also incorporated weighting to adjust for initial non-response and attrition, reducing the risk of bias which could result from these. In contrast with much of the existing literature we specifically considered our exposures as transitions between waves, and considered losses and gains separately, which allows for easier interpretation of findings and exploration of the possibility that positive and negative effects of the same exposure may differ. The use of fixed-effects analysis allowed us to eliminate the influence of any time invariant confounders, and careful consideration was taken to determine which time-varying confounders to include based on comprehensive literature searching. We presented a range of models to show the effects of each transition variable without adjustment and mutually adjusted for the other two variable groups, and also included sensitivity analyses to test the robustness of our modelling approach. Finally, we determined *a priori* a range of stratified analyses to report based on existing evidence of possible effect modifiers.

However, there are some limitations to our approach which should be acknowledged. With all panel data, non-response and attrition will increase over time, and while we attempted to overcome this using weights there remains a risk of bias or reduced external validity of findings. Issues with small sample size on stratification has led to poor precision around some estimates. It is difficult when data are collected annually to know for certain when one exposure happened in relation to another, which can pose challenges in differentiating between exposures and mediators. Our choice to include only lagged versions of the time-varying confounders should reduce the likelihood of inadvertently controlling for a mediator which is not one of our key variables of interest, but this does remain a risk with our mutually adjusted models which include both income and employment transitions. This is why we presented findings from Models 1, 2 and 3 side by side to allow readers to directly compare effect sizes for the transition variables between models. Also, though our use of transition variables improves ease of interpretation, there remains important nuance which is not captured by these, particularly around which elements of job loss or gain might be most important for mental health such as job security, job satisfaction or work/life balance.

Finally, while our fixed-effects approach aimed to estimate causal effects, causal inference is based on assumptions regarding the direction and timing of causal relationships between analysed variables. For example, lagged measures of potential confounders were treated as confounders, when they may in some instances have been acting as mediators for the effects of initial income/employment states (Petersen & van der Laan, 2014). Time-varying confounding of this nature is difficult to fully address in fixed-effects analyses and may inappropriately over-adjust for some of the potential mediating effects of the exposures we are interested in. This was a deliberate decision to allow us to more easily explore the influence of each of the three exposures of interest in parallel, but future work may triangulate results from alternative counterfactual-based approaches for single exposures e.g. calculating an average treatment effect using marginal structural modelling or g-computation (Naimi et al., 2016). Such approaches can more easily deal with time-varying confounding, but generally make stronger assumptions about no unmeasured confounding at the individual level than fixed-effects models.

### 4.4 Policy implications

Our research suggests that universal, one-size-fits-all policies aimed at raising income to the same degree for all households may not be sufficient to improve mental health for the UK population. Instead, more targeted policies focused on lifting people out of poverty may be more successful, particularly in reducing inequalities in mental health. This could either be achieved by increasing existing means-tested benefits such as Universal Credit (Royston, 2012), or by introducing new policies guaranteeing an income floor above the poverty line such as the Minimum Income for Healthy Living (Morris et al., 2000) or Universal Basic Income (Ruckert et al., 2018).

Our work also highlights that the mental health benefits of employment are considerably larger than income effects alone, so protecting people from unemployment should perhaps feature more prominently in discussions about income and welfare policy. The COVID-19 pandemic has demonstrated such an approach playing out on a grand scale, with the introduction of the UK's furlough scheme designed to protect both incomes and jobs during the associated economic downturn (Sunak, 2020). However, considerable care should be taken in translating these findings to practice under more ordinary circumstances. They should not, for example, be interpreted as an indication that simply increasing benefit conditionality to move unemployed individuals into unsatisfactory or temporary work will be beneficial for mental health: in fact, there is both qualitative (Wright et al., 2018) and quantitative (Katikireddi et al., 2018) evidence from the UK suggesting this is not the case. Instead, we suggest further research and policy exploration of which aspects of work are most important for wellbeing and incorporation of this nuance into future policy decisions, alongside protection from poverty.

### 4.5 Areas for future research

We believe there is a place for more causally-informed epidemiological analyses of specific income and employment status transitions in observational data, using methods designed for the causal question in hand (Petersen & van der Laan, 2014). Also, to complement the many high quality natural experiment studies in this area, [e.g. Reeves et al., 2017] more prospective trials of policies which influence income and employment in high-income countries settings would be useful, particularly if poverty status is likely to be affected. Where this is difficult or impossible, modelling studies which incorporate elements of findings from observational data and quasi-experimental studies may be useful for additional triangulation (Katikireddi, 2019). Finally, as described above further exploration of potential differences in the relationship between poverty transitions and mental health by sex in different populations and settings would be welcome.

## 5 Conclusions

While income changes alone might not be as important as one might expect for mental health, their effects appear be intertwined with changes in poverty and employment status. Becoming newly unemployed or moving below the poverty line has a clear negative impact on mental health, and reversing the situations where they occur could improve population mental health.

Economic and welfare policies which directly affect people's chances of living in poverty or being employed are highly likely to affect population mental health and wellbeing in a meaningful way, and these consequences should be actively taken into consideration in planning. Taking a Health in All Policies or Wellbeing Economy perspective may be a useful mechanism for such an approach (Coscieme et al., 2019; Ollila, 2011). Finally, we believe that income and employment should be thought of as related rather than separate concepts by policymakers – reducing one to increase the likelihood of the other is counterintuitive, and may well result in unanticipated and potentially negative consequences for mental health.

## Author contributions

RT and SVK conceived the idea for the study. TS conducted the analysis, and TS and RT drafted the manuscript. All authors contributed to the study design, interpretation of the findings, and critical revision of the manuscript, and approved the final version of the paper. The authors declare no conflicts of interest.

## Funding

This work was supported by the 10.13039/501100000724Health Foundation (grant number 2135162), the 10.13039/100010269Wellcome Trust (218105/Z/19/Z), NHS Research Scotland (SCAF/15/02), the 10.13039/501100000265Medical Research Council (MC_UU_00022/2), the 10.13039/501100000589Chief Scientist Office (SPHSU17) and the 10.13039/501100000781European Research Council (949582). There are no additional financial disclosures.
