# Supplementary material for: Which is most important for mental health: Money, poverty, or paid work? A fixed-effects analysis of the UK Household Longitudinal Study
Source: SSM Popul Health. 2021 Sep 4;15:100909. doi: 10.1016/j.ssmph.2021.100909 (PMC8455855; doi:10.1016/j.ssmph.2021.100909)
Supplement: Multimedia component 1 [file mmc1.docx]

# Appendix A: Model specification and weighting

**Regression equations**

The regression equation for Model 3 is as following:

${GHQ36}_{iw}=a_{0}+{\gamma X}_{iw}+\theta Z_{iw}+\mu_{i}+\pi_{w}+\varepsilon_{iw}$ *(S.1)*

where I represents individuals, w represents waves, α_ο_ is the intercept, γ represents the vector of our exposure variables, and θ represents a vector of all our additional covariates. The Greek letters μ and π represent individual and wave fixed-effects respectively, while ε is the error term that vary randomly across waves.

Models 3* and 3** are specified similarly with the difference that the outcome variable is a binary variable that shows whether the individual has a Common Mental Disorder or not (CMD in equation S.2)

${CMD}_{iw}=a_{0}+{\gamma X}_{iw}+\theta Z_{iw}+\mu_{i}+\pi_{w}+\varepsilon_{iw}$ *(S.2)*

**Use of fixed-effects vs random-effects**

This analysis uses a longitudinal dataset and therefore the choice between a fixed-effects or random-effects modelling strategy becomes apparent. We have chosen a fixed-effects approach for a number of reasons.

First and foremost, it is most suitable to test our research question. We are interested in investigating how mental health is affected by income change, poverty and employment status transitions within each individual across time. A fixed-effects approach allows for the individual specific effect to be correlated with explanatory variables. Since fixed-effects modelling demeans all variables in the model, it therefore produces estimates that reflect deviations from their means, and time invariant observable and unobservable variables are cancelled out, so omitted time invariant variables cannot bias results. However, we suspect that the average effect might be different by specific time-invariant variables such as sex, age and educational qualification groups, so we stratify our analysis using these three variables^[[1]](#footnote-1)^ to investigate how the average effect varies across these groups. The stratified models are presented in Table 3.

Moreover, the vast majority of empirical research considering the effect of income on mental health uses fixed-effects analysis, and therefore we apply the same technique to make our results comparable and also increase the potential of their replicability by future researchers.

We also applied two statistical tests to help us decide which model, fixed-effects or random-effects, is preferrable. First, we used the Sargan-Hansen statistic as a post estimation test after we ran Model 3 adjusted for random effects. This test is equivalent to a Hausman test, but unlike the Hausman test it is valid when the error terms are adjusted to be homoscedastic. It presented strong evidence against the null hypothesis that regressors are uncorrelated with group-specific errors, which is an assumption that needs to be met in order for a random-effects model to be valid. Second, we estimated a random-effects regression model adding group means, a technique known as the Mundlak technique,^[[2]](#footnote-2)^ which is also applicable when errors are adjusted to be homoscedastic. We then performed a joint Wald test for the whole model where the null hypothesis is that time-invariant unobservable variables are unrelated with our regressors. The Wald test appeared to be significant, which means that we can reject the null hypothesis and therefore the fixed-effect specification is preferable.

**Use of linear probability model vs fixed-effects logistic regression**

In order to estimate the relationship between income change, poverty status and employment status transitions and likelihood of common mental disorders (CMD) we used a Linear Probability Model (LPM) in a fixed-effects form, which in essence is a linear model with a binary outcome variable. Even if predicted OLS point estimates β remain unbiased estimates of the true parameter values β, we acknowledge the limitations of a LPM specification, such as the model suffering from inherent heteroskedasticity that leads to inconsistent standard errors, inaccurate p-values, also because of the non-normality of the dependent variable, and therefore to insufficient coefficient estimates, which in some cases can also become unrealistic predicted probabilities figures that can be lower than 0 and greater than 1.^[[3]](#footnote-3)^ However, the alternative choice of a fixed-effects logistic regression approach has other limitations: in particular that, by definition, the model would drop all time invariant observations. Therefore, in our case, participants with a consistent value in our outcome variable across all waves would have been excluded. Presenting this loss of power in numbers, we would lose 95,215 observations compared to using an LPM specification, which translates to 54.8% percent of observations in our sample. In terms of households, the loss accounts to 25,904 households, which translates to 66.9% of our total number of households in our sample. Since our analysis involves stratification the number of observations is vital, and fixed-effects logistic regression was therefore unsuitable to use.

Even though the gold standard for models with a binary outcome is logistic regression, LPM can perform equally well using remedies produced for each of these limitations, such as using robust standard errors, or variants of the LPM such as the Linear Discriminant Model (LDM) to force probabilities to lie within a (0,1) interval.^[[4]](#footnote-4),^^[[5]](#footnote-5)^ We applied all remedies apart from the LDM, as we did not encounter serious issues with unrealistic probabilities figures -only 175 observations had negative probabilities and just one a probability score higher than 1. However, to check that our choice did not markedly influence our results we ran a sensitivity analysis comparing the LPM and a conditional logit model using the restricted conditional logit sample and the same covariates as in our primary analysis.^[[6]](#footnote-6)^ The correlation between the predicted probabilities calculated in the two models is very high (0.97), indicating that LPM performs equally well to logistic regression. There is also relevant literature in support of using LPM with fixed effects over maximum likelihood specifications, ^[[7]](#footnote-7)^ with some arguing that this is even preferable when the number of observed CMD events in the outcome data is less than 25%.^[[8]](#footnote-8)^

**Weighting**

First, we calculated inverse probability weights (based on age, gender, ethnicity and education) within each wave to account for missing data in our outcome variable. Then, for each pair of survey waves (Wave_t-1_ and Wave_t_), we constructed an inverse-probability weight for having complete data for analysis across t-1 and t based on age in three age bands (18/29, 29/44, 45/65), Sex (male, female), Ethnicity (white, non-white), educational attainment (Degree/Other higher, A-Level, GCSE, None/Other), Government office region, the binary version of our outcome (GHQ) at Wave_t-1_ . This was combined with the published cross-sectional attrition/non-response weight for t-1 to obtain the final analytical weight used for each pair of waves in the analysis.

To investigate the potential impact of missing data on our findings, we compared the results from our primary analyses using these weights (which accounted for item missingness as described above) with a sensitivity analysis correcting only for non-response. The coefficients and confidence intervals produced were extremely similar, and there were no differences in the statistical inferences which could be made from each set of results.

# Appendix B: Flowchart of sample derivation

**Figure B1: Study flowchart in line with STROBE (Strengthening the Reporting of Observational Studies in Epidemiology) statement (http://www.strobestatement.org)**

Usoc observations (n) and participants (N) at baseline (Waves 1 to 10)
(n = 444,181; N=87,045)

Eligible Usoc observations and participants with full interview outcome

(n=415,678; N=81,875)

(n = 502,506)

Observations and participants interviewed as proxies
(n =28,503; N=5,170)

Eligible Usoc observations and participants who are adults and in working age (18-65y old)

(n=317,802; N=67,494)

Observations and participants aged less than 18 and more than 65 y old
(n =97876; N=14,381)

Eligible Usoc observations and participants who have a valid cross-sectional weight and non-response weight

(n=178,659; N=39,144)

Observations and participants with no valid weights
(n =139,143; N=28,350)

Eligible Usoc observations and participants who have no missing values in key variables

(n=173,859, N=38,697)

Observations and participants who have zero missing values in key variable
(n =4,800; N=447)

# Appendix C: Additional tables

**Table C1: No of observations and individuals with non-missing values in key variables across the whole sample**

|  | **Observations** | | **Individuals** | |
| --- | --- | --- | --- | --- |
|  | **Freq.** | **%** | **Freq.** | **%** |
| **One time** | 5,709 | 3.28 | 5,709 | 14.75 |
| **Two times** | 5,949 | 3.42 | 3,101 | 8.01 |
| **Three times** | 7,205 | 4.14 | 2,786 | 7.2 |
| **Four times** | 9,994 | 5.75 | 2,979 | 7.7 |
| **Five times** | 9,719 | 5.59 | 2,436 | 6.3 |
| **Six times** | 11,121 | 6.4 | 2,372 | 6.13 |
| **Seven times** | 20,691 | 11.9 | 3,851 | 9.95 |
| **Eight times** | 35,189 | 20.24 | 5,546 | 14.33 |
| **Nine times** | 68,282 | 39.27 | 9,917 | 25.63 |
| **Total** | **173,859** | **100,00** | **38,697** | **100.00** |

**Table C2: Sample statistics for continuous variables used in unstratified regression models**

|  | **Variable** |  | **Mean** | **Std. Dev.** | **Min** | **Max** | **Observations** |
| --- | --- | --- | --- | --- | --- | --- | --- |
| **Outcome** | **Outcome as continuous (GHQ-36)** | Overall | 11.29 | 5.63 | 0.00 | 36.00 | N = 173,875 |
|  |  | Between |  | 4.79 | 0.00 | 36.00 | n = 38,695 |
|  |  | within |  | 3.54 | -11.71 | 37.07 | **T-Bar= 4.49** |
| **Exposure** | **Income decreases (continuous)** | Overall | 6,999.86 | 32120.79 | 0.00 | 2,145,327.00 | N = 75,883 |
|  |  | Between |  | 28313.65 | 0.00 | 2,041,064.00 | n = 31,652 |
|  |  | Within |  | 22358.30 | -1,008,691.00 | 1,577,818.00 | **T-Bar= 2.40** |
|  | **Income increases (continuous)** | Overall | 5,906.56 | 20,123.48 | 0.00 | 2,246,255.00 | N = 97,976 |
|  |  | Between |  | 17,959.38 | 0.05 | 1,921,652.00 | n = 34,400 |
|  |  | Within |  | 14,438.46 | -1,085,996.00 | 1,341,373.00 | **T-Bar= 2.85** |
| **Covariates** | **Income (t-1)** | Overall | 16247.95 | 22024.88 | -24401.32 | 2396248 | N = 173,859 |
|  |  | Between |  | 15557.16 | -24401.32 | 966565.3 | n = 38,697 |
|  |  | Within |  | 16261.08 | -941028.1 | 1869017 | **T-Bar= 4.49** |
|  | **SF-12 Mental Component Summary (MCS) (t-1)** | Overall | 48.94 | 9.91 | 0 | 77.09 | N = 173,859 |
|  |  | Between |  | 8.57 | 0 | 74.4 | n = 38,697 |
|  |  | Within |  | 6.06 | 5.24 | 82.68 | **T-Bar= 4.49** |
|  | **SF-12 Physical Component Summary (PCS) (t-1)** | Overall | 51.28 | 10.04 | 4.48 | 76.29 | N = 173,859 |
|  |  | between |  | 9.11 | 5.28 | 74.46 | n = 38,697 |
|  |  | Within |  | 5.15 | 9.07 | 86.49 | **T-Bar= 4.49** |
|  | **Age** | Overall | 44.26 | 12.77 | 18 | 65 | N = 173,859 |
|  |  | between |  | 13.97 | 18 | 65 | n = 38,697 |
|  |  | Within |  | 2.06 | 19.25 | 69.00 | **T-Bar= 4.49** |

**Table C3: Effect of all economic activity transitions (Models 3, 3* and 3**)**

|  | **Model 3** | | | | **Model 3*** | | | | **Model 3**** | | | |
| --- | --- | --- | --- | --- | --- | --- | --- | --- | --- | --- | --- | --- |
|  | **Coef.** | **P>t** | **95% CI** | | **Coef.** | **P>t** | **95% CI** | | **Coef.** | **P>t** | **95% CI** | |
|  |  |  | **Lower** | **Higher** |  |  | **Lower** | **Higher** |  |  | **Lower** | **Higher** |
| **Employment in two consecutive waves*** | -1.460 | 0.000 | -1.874 | -1.045 | -0.095 | 0.000 | -0.125 | -0.066 | -0.117 | 0.000 | -0.148 | -0.086 |
| **Employment to Unemployment** | 2.061 | 0.000 | 1.805 | 2.317 | 0.158 | 0.000 | 0.136 | 0.180 | 0.174 | 0.000 | 0.152 | 0.197 |
| **Employment to inactivity** | -0.077 | 0.515 | -0.337 | 0.184 | 0.010 | 0.150 | -0.005 | 0.025 | 0.007 | 0.477 | -0.014 | 0.028 |
| **Employment to Long-Term disability** | 4.666 | 0.000 | 3.937 | 5.394 | 0.279 | 0.000 | 0.235 | 0.322 | 0.274 | 0.000 | 0.229 | 0.318 |
| **Unemployment to Employment** | -1.946 | 0.000 | -2.381 | -1.510 | -0.114 | 0.000 | -0.144 | -0.084 | -0.132 | 0.000 | -0.169 | -0.095 |
| **Unemployment in two consecutive waves**** | 1.460 | 0.000 | 1.045 | 1.874 | 0.095 | 0.000 | 0.066 | 0.125 | 0.117 | 0.000 | 0.086 | 0.148 |
| **Unemployment to Inactivity** | -1.045 | 0.000 | -1.455 | -0.634 | -0.076 | 0.004 | -0.118 | -0.033 | -0.086 | 0.000 | -0.120 | -0.051 |
| **Unemployment to Long-term disability** | 1.284 | 0.018 | 0.284 | 2.284 | 0.083 | 0.024 | 0.014 | 0.151 | 0.090 | 0.009 | 0.029 | 0.151 |
| **Inactivity to Employment** | -0.490 | 0.001 | -0.694 | -0.287 | -0.017 | 0.050 | -0.033 | 0.000 | -0.028 | 0.004 | -0.044 | -0.012 |
| **Inactivity to Unemployment** | 0.821 | 0.000 | 0.508 | 1.135 | 0.049 | 0.004 | 0.021 | 0.077 | 0.062 | 0.001 | 0.034 | 0.089 |
| **Inactivity in two consecutive waves**** | 0.155 | 0.159 | -0.075 | 0.385 | 0.008 | 0.324 | -0.010 | 0.026 | 0.014 | 0.153 | -0.006 | 0.035 |
| **Inactivity to Long-Term disability** | 2.222 | 0.000 | 1.643 | 2.801 | 0.124 | 0.000 | 0.076 | 0.173 | 0.138 | 0.000 | 0.083 | 0.194 |
| **Long-term disability to Employment** | -3.386 | 0.000 | -4.493 | -2.279 | -0.222 | 0.000 | -0.294 | -0.149 | -0.199 | 0.000 | -0.273 | -0.124 |
| **Long-term disability to Unemployment** | -0.644 | 0.099 | -1.439 | 0.151 | -0.031 | 0.283 | -0.093 | 0.031 | -0.013 | 0.659 | -0.079 | 0.053 |
| **Long-term disability to Inactivity** | -1.987 | 0.000 | -2.374 | -1.600 | -0.127 | 0.000 | -0.177 | -0.078 | -0.108 | 0.000 | -0.150 | -0.065 |
| **Long-term disability in two consecutive waves**** | 2.523 | 0.000 | 1.987 | 3.059 | 0.151 | 0.000 | 0.102 | 0.201 | 0.152 | 0.000 | 0.102 | 0.203 |

* The coefficient was estimated relative to unemployment in two consecutive waves

** The coefficient was estimated relative to employment in two consecutive waves

**Table C4: Effects of income, poverty & employment transitions on GHQ-36 score (Model 3) & likelihood of Common Mental Disorder (Models 3*[GHQ ≥ 4] & 3** [GHQ ≥ 3]) stratified by sex, age and education**

| **Exposures** | | | **10% Income change** | | | | | | | | **Poverty transitions** | | | | | | | | **Employment transitions** | | | | | | | **Statistics** | |
| --- | --- | --- | --- | --- | --- | --- | --- | --- | --- | --- | --- | --- | --- | --- | --- | --- | --- | --- | --- | --- | --- | --- | --- | --- | --- | --- | --- |
| **Transition type** | | | **Income decrease** | | | | **Income increase** | | | | **Into poverty** | | | **Out of poverty** | | | **Persisting poverty** | | **Into unemployment** | | | **Out of unemployment** | | **Persisting unemployment** | | **Obs** | **Clusters** |
| **Models** | | | **b** | | **P>\|t\|** | | **b** | | **P>\|t\|** | | **b** | | **P>\|t\|** | **b** | | **P>\|t\|** | **b** | **P>\|t\|** | **b** | **P>\|t\|** | | **b** | **P>\|t\|** | **b** | **P>\|t\|** |  | **(Individuals)** |
|  |  |  | **ci95%** | | | | **ci95%** | | | | **ci95%** | | | **ci95%** | | | **ci95%** | | **ci95%** | | | **ci95%** | | **ci95%** | |  | **(periods)** |
| **Full** | **Unstratified Model** | **3** | **0.004** | **0.051** | | **0.000** | | **0.143** | | **0.318** | | **0.007** | | **-0.360** | **0.000** | | **0.378** | **0.001** | **2.061** | **0.000** | **-1.946** | | **0.000** | **1.460** | **0.000** | **173,859** | **(38,697)**  **(9)** |
|  |  |  | **0, 0.007** | | | **-0.005, 0.001** | | | | **0.113, 0.523** | | | | **-0.49, -0.229** | | | **0.203, 0.553** | | **1.805, 2.317** | | **-2.381, -1.51** | | | **1.045, 1.874** | |  |  |
|  | **LPM** | **3*** | **0.000** | **0.081** | | **0.000** | | **0.510** | | **0.018** | | **0.034** | | **-0.018** | **0.022** | | **0.024** | **0.022** | **0.158** | **0.000** | **-0.114** | | **0.000** | **0.095** | **0.000** |  |  |
|  |  | **3**** | **0.000** | **0.051** | | **0.000** | | **0.000** | | **0.014** | | **0.082** | | **-0.018** | **0.017** | | **0.018** | **0.083** | **0.174** | **0.000** | **-0.132** | | **0.000** | **0.117** | **0.000** |  |  |
| **Sex** | **Male** | **3** | 0.004 | 0.028 | | -0.001 | | 0.064 | | 0.246 | | 0.061 | | -0.385 | 0.013 | | 0.404 | 0.016 | 2.199 | 0.000 | -1.989 | | 0.000 | 1.691 | 0.000 | **74,923** | **(17,139)**  **(9)** |
|  |  |  | 0.001, 0.008 | | | -0.057, 0.037 | | | | -0.014, 0.507 | | | | -0.663, -0.107 | | | 0.097, 0.711 | | 1.788, 2.609 | | -2.408, -1.571 | | | 1.26, 2.121 | |  |  |
|  |  | **3*** | 0.000 | 0.008 | | 0.000 | | 0.016 | | 0.012 | | 0.273 | | -0.020 | 0.087 | | 0.028 | 0.050 | 0.178 | 0.000 | -0.127 | | 0.000 | 0.112 | 0.000 |  |  |
|  |  | **3**** | 0.000 | 0.013 | | 0.000 | | 0.023 | | 0.014 | | 0.185 | | -0.017 | 0.115 | | 0.018 | 0.167 | 0.191 | 0.000 | -0.137 | | 0.000 | 0.136 | 0.000 |  |  |
|  | **Female** | **3** | 0.003 | 0.262 | | -0.003 | | 0.009 | | 0.380 | | 0.007 | | -0.343 | 0.001 | | 0.366 | 0.013 | 1.907 | 0.000 | -1.922 | | 0.000 | 1.191 | 0.004 | **98,936** | **(21,576)**  **(9)** |
|  |  |  | -0.003, 0.008 | | | -0.063, 0.006 | | | | 0.135, 0.625 | | | | -0.507, -0.18 | | | 0.1, 0.632 | | 1.355, 2.458 | | -2.665, -1.178 | | | 0.503, 1.879 | |  |  |
|  |  | **3*** | 0.000 | 0.056 | | 0.000 | | 0.005 | | 0.023 | | 0.008 | | -0.016 | 0.047 | | 0.021 | 0.029 | 0.135 | 0.000 | -0.103 | | 0.001 | 0.082 | 0.007 |  |  |
|  |  | **3**** | 0.000 | 0.021 | | 0.000 | | 0.005 | | 0.014 | | 0.085 | | -0.019 | 0.008 | | 0.017 | 0.084 | 0.155 | 0.000 | -0.136 | | 0.001 | 0.103 | 0.002 |  |  |
| **Age group** | **18/29** | **3** | 0.004 | 0.304 | | 0.000 | | 0.094 | | 0.137 | | 0.458 | | -0.460 | 0.043 | | 0.367 | 0.049 | 2.136 | 0.000 | -1.667 | | 0.002 | 1.041 | 0.022 | **27,763** | **(9,816)**  **(9)** |
|  |  |  | -0.005, 0.014 | | | -0.09, 0.084 | | | | -0.268, 0.542 | | | | -0.904, -0.017 | | | 0.003, 0.732 | | 1.278, 2.994 | | -2.526, -0.808 | | | 0.194, 1.888 | |  |  |
|  |  | **3*** | 0.001 | 0.013 | | 0.000 | | 0.035 | | 0.000 | | 0.974 | | -0.034 | 0.031 | | 0.021 | 0.109 | 0.160 | 0.000 | -0.095 | | 0.006 | 0.074 | 0.014 |  |  |
|  |  | **3**** | 0.001 | 0.015 | | 0.000 | | 0.029 | | 0.003 | | 0.786 | | -0.033 | 0.116 | | 0.014 | 0.464 | 0.181 | 0.000 | -0.133 | | 0.006 | 0.120 | 0.007 |  |  |
|  | **29/44** | **3** | 0.000 | 0.859 | | -0.004 | | 0.007 | | 0.406 | | 0.036 | | -0.273 | 0.070 | | 0.333 | 0.089 | 1.738 | 0.000 | -2.030 | | 0.000 | 1.930 | 0.001 | **55,957** | **(14,772)**  **(9)** |
|  |  |  | -0.007, 0.006 | | | -0.09, 0.005 | | | | 0.034, 0.777 | | | | -0.575, 0.029 | | | -0.064, 0.731 | | 1.183, 2.294 | | -2.842, -1.217 | | | 1.137, 2.723 | |  |  |
|  |  | **3*** | 0.000 | 0.021 | | 0.000 | | 0.004 | | 0.025 | | 0.078 | | -0.006 | 0.583 | | 0.026 | 0.125 | 0.138 | 0.000 | -0.124 | | 0.000 | 0.121 | 0.002 |  |  |
|  |  | **3**** | 0.000 | 0.032 | | 0.000 | | 0.043 | | 0.023 | | 0.050 | | -0.012 | 0.355 | | 0.021 | 0.213 | 0.152 | 0.000 | -0.130 | | 0.001 | 0.129 | 0.001 |  |  |
|  | **45/65** | **3** | 0.004 | 0.048 | | -0.002 | | 0.035 | | 0.390 | | 0.007 | | -0.436 | 0.006 | | 0.485 | 0.002 | 2.179 | 0.000 | -2.206 | | 0.000 | 1.809 | 0.000 | **90,171** | **(20,414)**  **(9)** |
|  |  |  | 0, 0.009 | | | -0.064, 0.026 | | | | 0.144, 0.637 | | | | -0.71, -0.162 | | | 0.233, 0.737 | | 1.666, 2.691 | | -2.733, -1.678 | | | 1.205, 2.414 | |  |  |
|  |  | **3*** | 0.000 | 0.012 | | 0.000 | | 0.058 | | 0.025 | | 0.005 | | -0.023 | 0.036 | | 0.028 | 0.010 | 0.165 | 0.000 | -0.124 | | 0.000 | 0.108 | 0.000 |  |  |
|  |  | **3**** | 0.000 | 0.001 | | 0.000 | | 0.051 | | 0.019 | | 0.009 | | -0.023 | 0.029 | | 0.025 | 0.018 | 0.183 | 0.000 | -0.127 | | 0.000 | 0.121 | 0.001 |  |  |
| **Educational Qualifications** | **Degree/Other Higher** | **3** | 0.002 | 0.328 | | -0.004 | | 0.002 | | 0.264 | | 0.095 | | -0.374 | 0.023 | | 0.452 | 0.011 | 1.942 | 0.000 | -1.718 | | 0.001 | 1.125 | 0.008 | **75,472** | **(15,734)**  **(9)** |
|  |  |  | -0.003, 0.008 | | | -0.068, -0.006 | | | | -0.057, 0.585 | | | | -0.681, -0.067 | | | 0.138, 0.767 | | 1.498, 2.385 | | -2.426, -1.01 | | | 0.388, 1.862 | |  |  |
|  |  | **3*** | 0.000 | 0.024 | | 0.000 | | 0.008 | | 0.016 | | 0.178 | | -0.027 | 0.053 | | 0.036 | 0.035 | 0.158 | 0.000 | -0.078 | | 0.012 | 0.051 | 0.034 |  |  |
|  |  | **3**** | 0.000 | 0.051 | | 0.000 | | 0.032 | | 0.010 | | 0.398 | | -0.028 | 0.034 | | 0.028 | 0.111 | 0.198 | 0.000 | -0.110 | | 0.000 | 0.092 | 0.001 |  |  |
|  | **A-Level** | **3** | -0.001 | 0.689 | | -0.003 | | 0.028 | | 0.287 | | 0.010 | | -0.264 | 0.178 | | 0.107 | 0.529 | 2.290 | 0.000 | -2.490 | | 0.000 | 2.268 | 0.001 | **38,522** | **(9,887)**  **(9)** |
|  |  |  | -0.009, 0.006 | | | -0.088, 0.029 | | | | 0.09, 0.484 | | | | -0.675, 0.148 | | | -0.268, 0.483 | | 1.521, 3.06 | | -3.439, -1.542 | | | 1.318, 3.218 | |  |  |
|  |  | **3*** | 0.000 | 0.097 | | 0.000 | | 0.063 | | 0.028 | | 0.020 | | -0.017 | 0.298 | | 0.016 | 0.103 | 0.129 | 0.001 | -0.177 | | 0.002 | 0.168 | 0.002 |  |  |
|  |  | **3**** | 0.000 | 0.089 | | 0.000 | | 0.091 | | 0.017 | | 0.117 | | -0.016 | 0.298 | | 0.004 | 0.751 | 0.132 | 0.001 | -0.178 | | 0.002 | 0.165 | 0.002 |  |  |
|  | **GCSE** | **3** | 0.005 | 0.293 | | 0.001 | | 0.085 | | 0.424 | | 0.003 | | -0.273 | 0.126 | | 0.360 | 0.027 | 1.915 | 0.000 | -1.598 | | 0.001 | 1.263 | 0.002 | **35,933** | **(8,457)**  **(9)** |
|  |  |  | -0.005, 0.015 | | | -0.066, 0.078 | | | | 0.187, 0.661 | | | | -0.642, 0.095 | | | 0.052, 0.668 | | 1.193, 2.636 | | -2.299, -0.897 | | | 0.619, 1.906 | |  |  |
|  |  | **3*** | 0.000 | 0.026 | | 0.000 | | 0.092 | | 0.015 | | 0.138 | | -0.003 | 0.832 | | 0.013 | 0.448 | 0.183 | 0.000 | -0.097 | | 0.002 | 0.097 | 0.004 |  |  |
|  |  | **3**** | 0.000 | 0.021 | | 0.000 | | 0.053 | | 0.017 | | 0.199 | | -0.003 | 0.857 | | 0.013 | 0.442 | 0.192 | 0.000 | -0.122 | | 0.003 | 0.117 | 0.001 |  |  |
|  | **None/Other** | **3** | 0.012 | 0.013 | | 0.000 | | 0.100 | | 0.442 | | 0.120 | | -0.689 | 0.002 | | 0.740 | 0.001 | 2.390 | 0.000 | -2.305 | | 0.003 | 1.601 | 0.003 | **23,932** | **(6,369)**  **(9)** |
|  |  |  | 0.003, 0.021 | | | -0.081, 0.082 | | | | -0.144, 1.028 | | | | -1.045, -0.332 | | | 0.41, 1.069 | | 1.413, 3.366 | | -3.566, -1.045 | | | 0.702, 2.499 | |  |  |
|  |  | **3*** | 0.001 | 0.005 | | 0.000 | | 0.090 | | 0.019 | | 0.245 | | -0.029 | 0.048 | | 0.033 | 0.022 | 0.169 | 0.002 | -0.119 | | 0.003 | 0.095 | 0.003 |  |  |
|  |  | **3**** | 0.001 | 0.001 | | 0.000 | | 0.040 | | 0.022 | | 0.238 | | -0.030 | 0.098 | | 0.029 | 0.062 | 0.156 | 0.002 | -0.129 | | 0.008 | 0.113 | 0.001 |  |  |

|  | p≥0.05 |  | 0.05>p≥0.010 |  | p<0.010 |
| --- | --- | --- | --- | --- | --- |

# Appendix D: Additional graphs

**Figure D1: Fixed effects linear models (Models 1-3) - Relationship between income/poverty/employment transitions and GHQ-36 score**

**
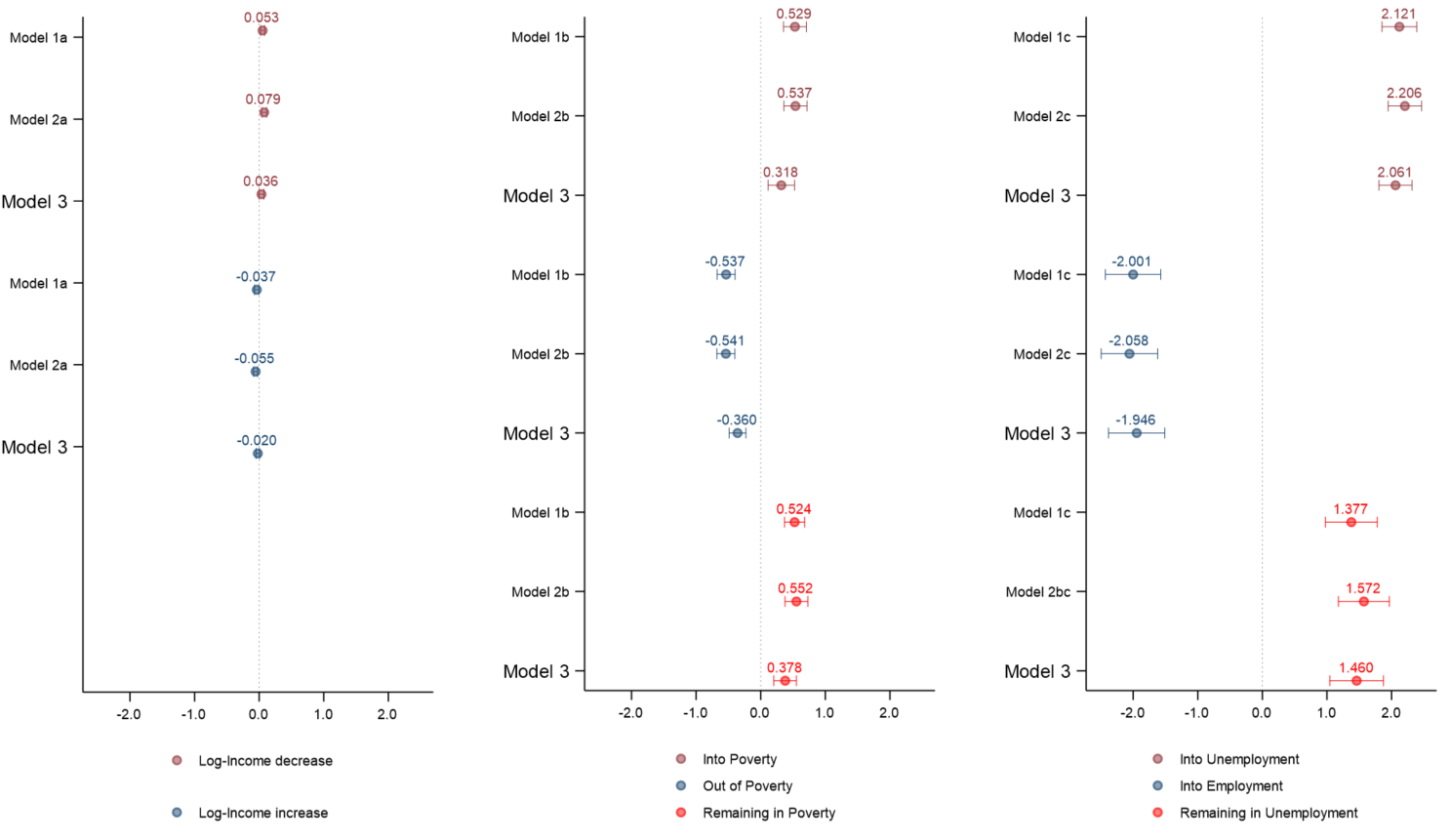
**

**Figure D2: Fixed-effects linear probability models (Model 3* (Specification 1) and 3** (Specification 2) - Relationship between income/poverty/employment transitions and likelihood of common mental disorder**

**
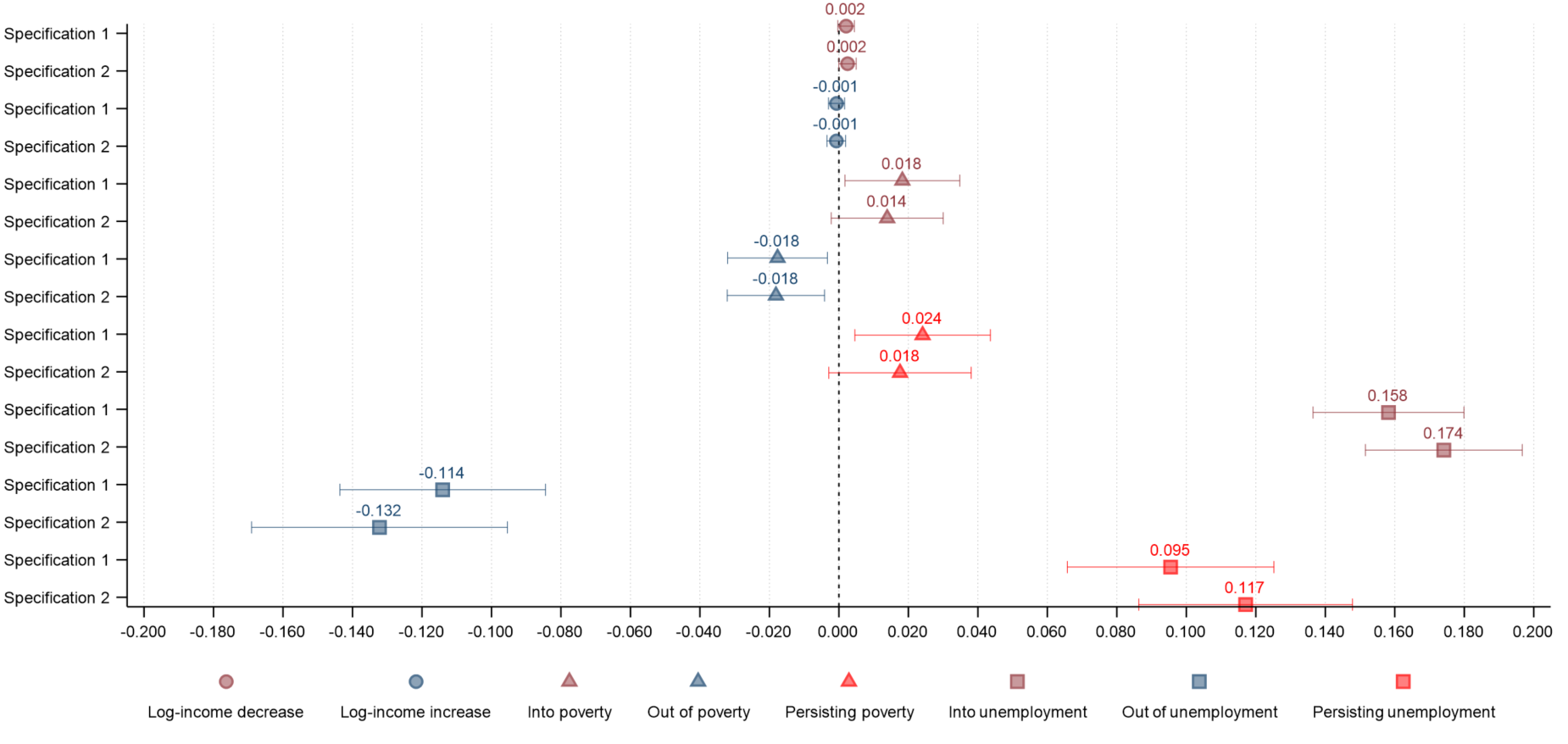
**

**Figure D3: Fixed-effects linear models (Model 3) - Relationship between income/poverty/employment transitions and GHQ-36 score - stratified by sex, age group and highest education**

**
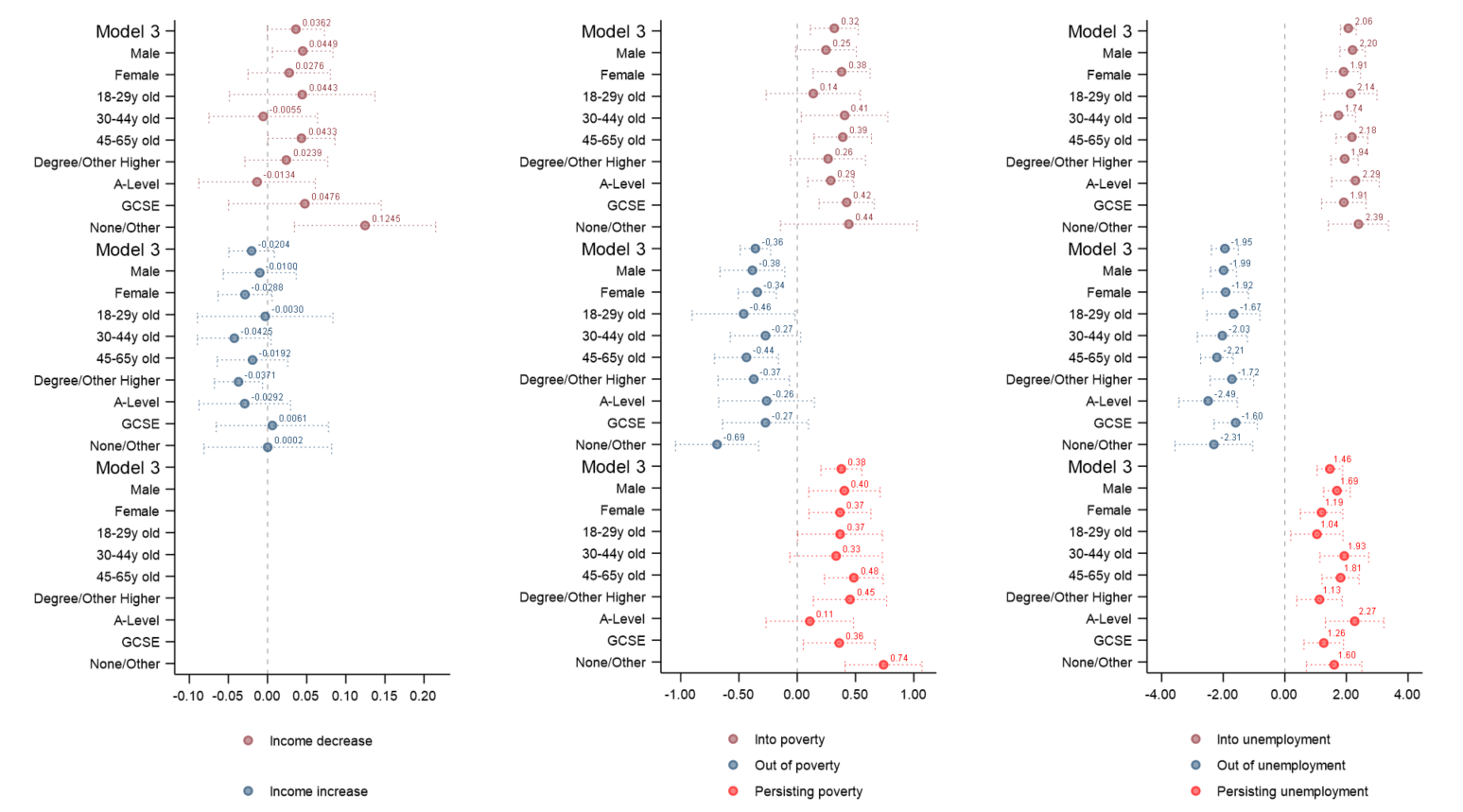
**

**Figure D4: Fixed-effects linear probability models (Model 3*) - Relationship between income/poverty/employment transitions and likelihood of common mental disorder - stratified by sex, age group and highest education**


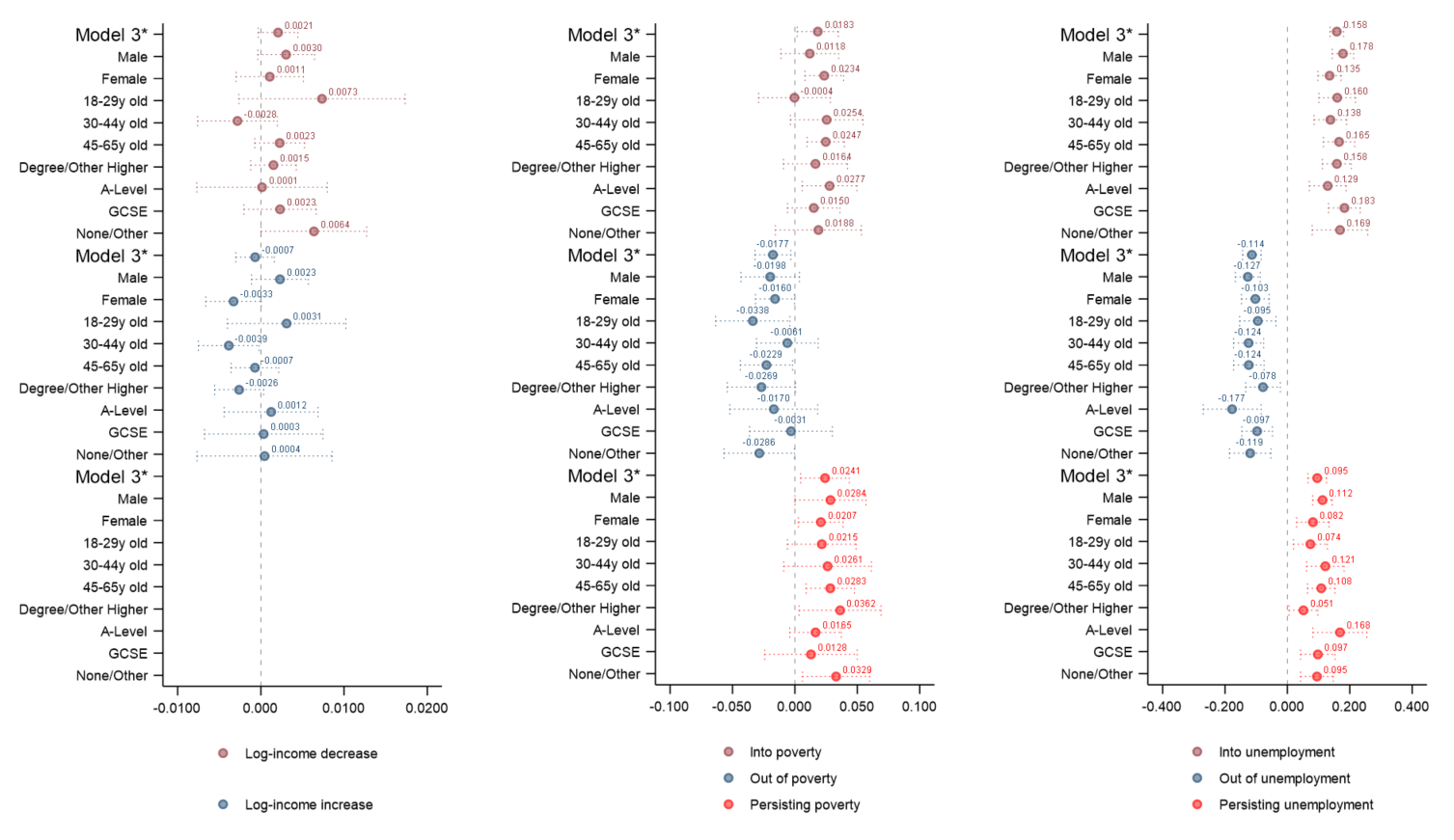


1. Because our sample includes younger individuals (>18y old) there is some limited within subject variation with educational qualifications and therefore we have decided to include it in the fixed-effect equation in Models 2 and 3 as a control variable. [↑](#footnote-ref-1)
2. Mundlak, Y. (1978) "On the Pooling of Time Series and Cross-section Data" Econometrica, 46: 69–85. [↑](#footnote-ref-2)
3. Aldrich, J. H., & Nelson, F. D. (1984). Linear probability, logit, and probit models (No. 45). Sage. [↑](#footnote-ref-3)
4. Zhao, L., Chen, Y., & Schaffner, D. W. (2001). Comparison of logistic regression and linear regression in modeling percentage data. Applied and environmental microbiology, 67(5), 2129-2135. [↑](#footnote-ref-4)
5. Haggstrom, G. W. (1983). Logistic regression and discriminant analysis by ordinary least squares.Journal of Business & Economic Statistics,1(3), 229-238 [↑](#footnote-ref-5)
6. Junna L, Moustgaard H, Tarkiainen L, Martikainen P. The association between income and psychotropic drug purchases: Individual fixed effects analysis of annual longitudinal data in 2003–2013. Epidemiology. 2019 Mar 1;30(2):221-9. [↑](#footnote-ref-6)
7. Beck N. Estimating grouped data models with a binary-dependent variable and fixed effects via a logit versus a linear probability model: The impact of dropped units. Political Analysis. 2020 Jan;28(1):139-45. [↑](#footnote-ref-7)
8. Timoneda JC. Estimating group fixed effects in panel data with a binary dependent variable: how the LPM outperforms logistic regression in rare events data. Social Science Research. 2021 Jan 1;93:102486. [↑](#footnote-ref-8)
